# *Trypanosoma tertium* n. sp.: prevalences in natural hosts and development in the mosquito vector

**DOI:** 10.1017/S0031182025000587

**Published:** 2025-04

**Authors:** Magdaléna Kulich Fialová, Anna Kapustová, Ivan Čepička, Milena Svobodová

**Affiliations:** 1 Department of Parasitology, Faculty of Science, Charles University, Prague, Czech Republic; 2 Department of Zoology, Faculty of Science, Charles University, Prague, Czech Republic

**Keywords:** avian blood parasite, *Culex*, monoxenous Kinetoplastea, morphology, phylogeny, transmission, vector

## Abstract

Avian trypanosomes (Trypanosoma, Kinetoplastea) are successful blood parasites occurring worldwide. These parasites are usually non-pathogenic to their avian hosts, thus neglected in studies regarding their life cycles and vectors. Several families of blood-sucking dipteran insects, including mosquitoes, have been identified as vectors of avian trypanosomes. Mosquitoes have been experimentally confirmed as vectors of *Trypanosoma culicavium* and *Trypanosoma thomasbancrofti*. In this study, we describe a third species of avian trypanosomes occurring in mosquitoes, designated as *Trypanosoma tertium* n. sp. This species can be distinguished from related trypanosome species based on morphology and small subunit rRNA gene sequence. Two isolates of *T. tertium* n. sp. obtained from a mosquito and a bird host were able to infect two subspecies of laboratory *Culex pipiens* mosquitoes, with infection rates reaching 60% and heavy infections in 90% of positive females. In infected mosquitoes, trypanosomes occurred as long epimastigotes in the midgut and short epimastigotes and rosettes in the hindgut. Putative infectious stages were detected in the diuretic liquid of infected mosquitoes, suggesting, besides transmission through ingestion of the infected vector, a possible transconjunctival infection. Among wild mosquitoes, avian trypanosomes were detected exclusively in *Cx. pipiens* with 3.3% total prevalence, while *T. tertium* n. sp. prevalence was only 0.08% among 1128 dissected *Cx. pipiens* individuals. In birds, *T. tertium* n. sp. was detected in 8 species within which the prevalence was 1.3% (686 birds), while it was 0.3% in total (3084 birds). We discuss the relationship of the newly described *T. tertium* n. sp. with other mosquito-transmitted trypanosomes.

## Graphical abstract

**Figure:**
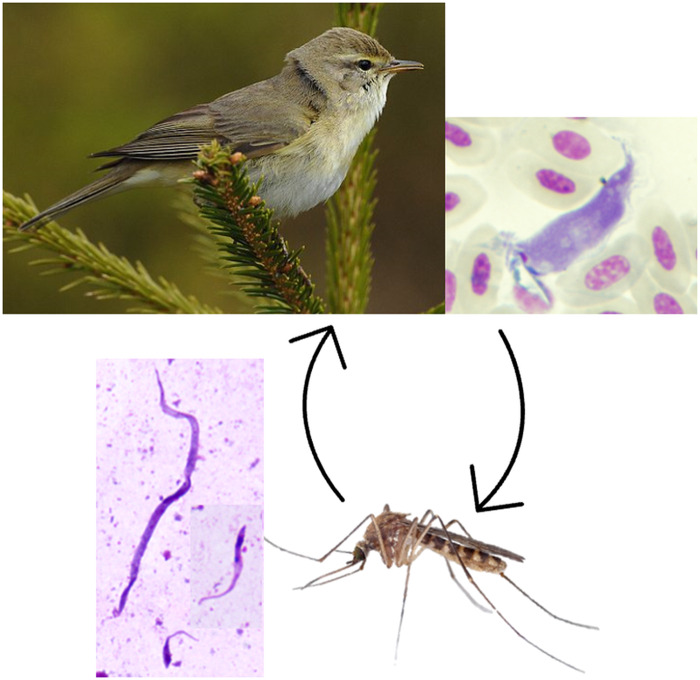

## Introduction

Protists of the genus *Trypanosoma* (Trypanosomatidae, Kinetoplastea) are dixeneous blood parasites transmitted by bloodsucking invertebrates. They are known for causing illnesses in humans and animals such as Chagas disease, sleeping sickness, nagana, etc. Trypanosomes also readily infect birds, with a relatively high prevalence (Bennett et al., 1974; Greiner et al., 1975; Sebaio et al., 2012; Zamora-Vilchis et al., 2012; Galen et al., 2020; Svobodová et al., 2023; Musa et al., 2024). However, due to their low health and economic impact, avian trypanosomes remain largely neglected.

Little attention is paid to the vectors of avian trypanosomes as well, despite their essential role in maintaining the parasites in the host populations. Various insect species have been identified as vectors of avian trypanosomes, including black flies (Simuliidae, Bennett, 1961; Votýpka et al., 2002; Votýpka and Svobodová, 2004), hippoboscid flies (Hippoboscidae, Baker, 1956; Votýpka et al., 2002; Santolíková et al., 2022), mosquitoes (Culicidae, Bennett, 1961; Votýpka et al., 2012; Fialová et al., 2021), biting midges (Ceratopogonidae, Miltgen and Landau, 1982; Svobodová et al., 2017; Bernotienė et al., 2020) and sand flies (Psychodidae, Kato et al., 2011; Svobodová and Rádrová, 2018). Avian trypanosome mature infections are localized in the intestine (midgut, hindgut) of their vectors, and the most common transmission mode to birds is peroral, by ingestion of an infected vector (Bennett, 1961; Votýpka and Svobodová, 2004; Votýpka et al., 2012; Svobodová and Rádrová, 2018; Fialová et al., 2021). An alternative way of transmission is through the conjunctiva, when metacyclic stages from the vector’s hindgut are expelled with the prediuretic/diuretic liquid while the vector feeds (Votýpka and Svobodová, 2004; Fialová et al., 2021).

Mosquitoes are notoriously associated with the transmission of the protozoan of the genus *Plasmodium,* the causative agents of malaria in reptiles, amphibians, mammals and birds. In contrast, their role in the life cycle of trypanosomes is less explored. However, as early as in 1843, mosquitoes were identified as vectors of *Trypanosoma rotatorium*, the species from anurans (Mayer, 1843 in Desser et al., 1973), and they were also among the first suspected vectors of avian trypanosomes (Novy et al., 1907; Bennett, 1961, 1970; Chatterjee, 1977). Presently, three species/lineages of avian trypanosomes infecting mosquitoes are recognized, namely: *Trypanosoma culicavium, Trypanosoma thomasbancrofti*, and *Trypanosoma* sp. lineage III (Votýpka et al., 2012; Zídková et al., 2012; Šlapeta et al., 2016; Fialová et al., 2021; Kulich Fialová, 2024). While *T. culicavium* and *T. thomasbancrofti* life cycles have been experimentally confirmed and the species have been formally described, the life cycle of the *Trypanosoma* sp. lineage III remains unknown as well as its characteristics have not been studied in detail.

Our objective was to characterize the least known avian trypanosome found in mosquitoes – *Trypanosoma* sp. lineage III. Together with its sister species *T. thomasbancrofti*, this trypanosome belongs to the group C of avian trypanosomes, which includes other *T. avium* s. l. lineages as well (Zídková et al., 2012). This study investigated the experimental life cycle and prevalence of an uncharacterized avian trypanosome in both avian hosts and mosquitoes. It provides new insights into its life cycle and morphological features, which were previously unknown, further expanding our understanding of mosquito-transmitted avian trypanosomes.

## Materials and methods

### Prevalence in naturally infected mosquitoes

Mosquitoes were trapped in July 2018–2021 in Milovice Forest, South Moravia, Czechia (48.8213 N, 16.6932 E), using CDC traps (JW Hock Company, Gainesville, FL, USA) with CO_2_ as an attractant. Mosquitoes were collected into nylon nets connected to the traps, anesthetized on ice and sorted according to species (Kramář, 1958). Individual mosquitoes were killed in 70% ethanol, rinsed twice in sterile saline and then dissected using tweezers (Dumont tweezers, Ted Pella, California, USA), which were sterilized in a flame between each dissection to prevent cross contamination. Dissected guts were examined under the light microscope for the presence of kinetoplastids. A part of each positive gut was used for the cultivation of kinetoplastids, and the rest was stored in ethanol for barcoding of the parasites (see below).

### Prevalence in wild birds

Bird sampling was done at several Czech localities in 2014–2016 between May and July. Adults and yearlings were mist-netted by the authors or in cooperation with other registered ringers contributing to the programme Constant Effort Site, organized by the Prague Ringing Centre. Trapping and sampling were done by licensed workers according to national law and experimental guidelines. Blood was collected as described in Fialová et al. (2021); a part was used for trypanosome cultivation; a part was stored in ethanol at −20 °C prior to further use. Blood smears on glass slides were prepared from each bird as well. After air-drying of the blood film, the smears were fixed in absolute methanol and stained with Giemsa for 30 min.

### Parasite strains and cultures

Both trypanosome strains used in this study originated from our own collection and were acquired in Milovice Forest: CUL5 was isolated from a *Culex pipiens* female (ICUL/CZ/2000/CUL5) and PAS416 from a willow warbler (*Phylloscopus trochilus*) (APHY/CZ/2016/PAS416). The isolate CUL5 was assigned to the lineage III of group C previously (Zídková et al., 2012; GenBank Acc. No. JN006838); PAS416 was barcoded by nested polymerase chain reaction (PCR) as described below. The small subunit (SSU) rRNA sequence comparison of PAS416 with CUL5 using the BLAST algorithm revealed 99.7% identity, while there were 2 differences in a 635 bases-long segment.

Trypanosomes were cultivated in flat tubes or PEN tubes (diagnostic cultivation) on blood agar (SNB-9; Diamond and Herman, 1954) prepared from rabbit (Bioveta, Ivanovice na Hané, Czech Republic) or sheep (LabMediaServis, Jaroměř, Czech Republic) blood and overlaid with liquid medium. Liquid medium was prepared by mixing 1:1 RPMI 1640 and Schneider’s *Drosophila* Medium (both from Sigma-Aldrich, St. Louis, MO, USA), supplemented with 10% FCS (Gibco, Thermo Fisher Scientific, Inc., Waltham, MA, USA), 2% sterile human urine and 50 μg/mL amikacin (Medochemie, Prague, Czech Republic). Media for isolation used in field studies were, moreover, supplemented with penicillin (10 000 IU/mL, BB Pharma a.s., Prague, Czechia) and fluorocytosine (1500 μg/mL, TCI, Tokyo, Japan). Thriving cultures from PEN tubes or flat tubes were subcultured into new flat tubes with fresh blood agar and overlay. Cultures were held at 23 °C.

### Experimental infections of mosquitoes

Two subspecies of *Cx. pipiens* were used in this study; both were bred in our laboratory: *Culex pipiens quinquefasciatus* (*Cx. p. quinquefasciatus* henceforth), originating from India and kept in our laboratory for more than 30 years, and *Culex pipiens molestus* (*Cx. p. molestus* henceforth), colonized from individuals caught in Czechia in 2016. A colony of *Aedes aegypti* was established temporarily from mosquitoes obtained from the National Institute of Public Health, Prague.

Mosquito females (8–11 days old) were infected by feeding through a chick skin membrane on a glass feeder containing inactivated (30 min in 56 °C water bath) sheep or rabbit blood mixed with 12–18-day-old cultures of trypanosomes (2–6 × 10^8^ parasite cells/mL). After each blood feeding, the blood fed to mosquitoes was controlled under the microscope for the presence of live trypanosomes. Fed females were separated and kept in nets in an incubator with stable conditions (21 °C, convenient humidity) and provided with a 50% sucrose solution on a cotton pad. Mosquitoes were dissected 10–45 days post-infection. Dissected guts were examined under the light microscope for infection status, intensity of infection, parasite localization and its changes during the course of infection. Infection intensities were defined as low, 1–100 parasites; medium, 100–1000 parasites; and heavy, >1000 parasites per gut.

In the experiment testing *Ae. aegypti* susceptibility, *Cx. p. quinquefasciatus* and *Ae. aegypti* mosquitoes were fed in parallel on the same blood meal with isolate CUL5. Five mosquitoes in each group were dissected 3 days after inoculation to control whether trypanosomes were transmitted during experimental feeding and were present in the ingested blood. The rest of the mosquitoes were dissected after blood ingestion as described above.

### Trypanosomes in diuretic liquid

Mosquitoes, membrane-fed on blood with strain PAS416, were provided with a bowl of water 4 days after feeding to allow oviposition. After oviposition, 10 days after first feeding, the mosquitoes were provided with an anesthetized laboratory mouse, BALB/c. The feeding was monitored, and females that finished blood feeding were transferred immediately into plastic *Drosophila* tubes with a coverslip placed on the bottom, which served to catch droplets of diuretic liquid. Air-dried droplets were fixed with methanol, stained with Giemsa and examined for the presence of trypanosomes. Mosquito females were dissected to assess their infection status.

### Experimental inoculation of birds

All experimental birds, originally purchased at a pet store, were screened for the presence of trypanosomes prior to inoculation. Screening was done with blood obtained from vein articulation (vena metatarsalis plantaris superficialis media), using cultivation (see above) and PCR screening (see below). All birds were negative.

Birds were inoculated with 7–10 *Cx. p. molestus* or *Cx. p. quinquefasciatus* infected guts homogenized in saline, applied orally, subcutaneously or by placing on the conjunctiva, i.e. transconjunctivally. Birds were tested for the presence of trypanosomes by blood cultivation at weekly intervals (4 weeks), then monthly (5 months); obtained cultures were checked microscopically 3 times at weekly intervals.

### PCR diagnostic

The High Pure PCR Template Preparation Kit (Roche Diagnostic, Mannheim, Germany) was used for DNA extraction. The obtained DNA was subsequently stored at −20 °C until further use. Amplification of the trypanosome SSU rRNA gene from bird blood and dissected guts of mosquitoes was done by specific nested PCR with primers S762/S763 for the first step (Maslov et al., 1996) and TR-F2/TR-R2 for the second step (Votýpka et al., 2015); cultivated trypanosomes established from mosquito guts were barcoded by single-step PCR with primers Medlin A/Medlin B (Medlin et al., 1988), as described in Fialová et al. (2021). Obtained PCR products were purified using the enzymatic solution ExoSap (Thermo Scientific, Waltham, MA, USA) and then sequenced at the core facility of the Faculty of Science (Laboratory OMICS – Genomics, Biocev). Sequences were examined in the BioEdit software and analysed using the BLAST algorithm and nucleotide database NCBI.

### Scanning electron microscopy

Guts of mosquitoes positive for trypanosomes after experimental infection were torn by insulin syringe, then fixed in 2.5% glutaraldehyde in 5 mM HCl, 0.1 M cacodylate buffer for 24 h at 4 °C. Thereafter, they were processed at our core facility, the Laboratory of Electron Microscopy, as follows: samples were post-fixed in 2% osmium tetroxide in the same buffer for 2 h at room temperature. After dehydration in a graded ethanol series, the guts were critical-point air-dried, sputter-coated with gold in a Polaron coater Bal-Tec SCD050 (Bal-Tec Ag, Balzers, Liechtenstein) and examined by the authors using a JEOL 6380LV (JEOL, Tokyo, Japan) scanning electron microscope.

### Light microscopy and measurement of trypanosomes

Positive guts of mosquitoes from experimental infections were fixed on slides with methanol, stained with Giemsa (Sigma-Aldrich, St. Louis, MO, USA) and photographed at 1000× magnification with a CDC camera (DP70) using an Olympus BX51 microscope. Measurement of the cells was done using ImageJ software. Data from the cell measurements were processed using R software (R Core Team, 2018) – the mean values of trypanosomes in mosquito gut and cultures (body length, body width, flagellum length) and their standard deviation were calculated as described in Crawley (2013).

Blood smears (fixed with methanol and stained with Giemsa) of birds positive by PCR for *Trypanosoma* sp. lineage III were inspected under the microscope at magnification 1000× for 10 min, and the whole smear area at 200× for unlimited time.

## Results

### Prevalence of Trypanosoma tertium n. sp. in wild-caught mosquitoes

In total, 2246 wild-caught mosquitoes were dissected; the majority of them were identified as *Cx. pipiens* (1128), and the rest were mosquitoes of the genera *Aedes* (*Ae. annulipes, Ae. cantans, Ae. caspius, Ae. cinereus, Ae. excrucians, Ae. punctor, Ae. rusticus, Ae. sticticus, Ae. vexans*), *Anopheles* (*An. claviger, An. maculipennis, An. plumbeus*), *Culiseta annulata* and *Mansonia richiardii*. All species of mosquitoes infected with kinetoplastids were investigated by PCR; however, mosquito-specific avian trypanosomes (*T. culicavium, T. tertium* n. sp., *T. thomasbancrofti*) were found exclusively in *Cx. pipiens*, with the prevalence of 3.3% from 1128 dissected individuals. Furthermore, *Culex* mosquitoes harboured mammalian trypanosomes belonging to *Trypanosoma theileri* s. l. as well as monoxenous kinetoplastids of the genus *Crithidia* and *Paratrypanosoma* (for parasite species and prevalences, see Table 1). *Trypanosoma theileri* s. l. was identified in *Aedes, Anopheles, Culiseta* and *Mansonia* spp., monoxenous kinetoplastids in *Aedes* and *Mansonia* spp. as well.

**Table 1.** Prevalences of trypanosomatids in dissected mosquito species and/or genera

Mosquito genus/species	No. dissected	*T. culicavium*	*T. tertium* n. sp.	*T. thomasbancrofti*	T. theileri^*^	C. brevicula^**^	C. dedva^***^	C. dobrovolskii^****^	*P. confusum*
*Aedes* spp.	959				157 (16.4%)	1 (0.1%)	1 (0.1 %)	3 (0.3 %)	
*Anopheles* spp.	33				1 (3%)				
*Cx. pipiens*	1128	36 (3.1%)	1 (0.08%)	1 (0.08%)	4 (0.3%)		1 (0.08 %)	2 (0.17 %)	8 (0.7%)
*Cs. annulata*	43				2 (4.6%)				
*Ms. richiardii*	83				1 (1.2%)	1 (1.2%)			

Found in the following species:

* *Ae. annulipes* (2/7), *Ae. cantans* (3/14), *Ae. excrucians* (3/19), *Ae. punctor* (11/27), *Ae. sticticus* (1/4), *Ae. vexans* (137/846) and *An. plumbeus* (1/30).

** *Ae. annulipes* (1/7).

*** *Ae. vexans* (1/846).

**** *Ae. caspius* (1/14)*, Ae. vexans* (2/846).

### Prevalence of Trypanosoma tertium n. sp. in wild-caught passerines

*Trypanosoma tertium* n. sp. was detected in 9 (0.3%) of the 3084 sampled individuals. Infected species included barn swallow (*Hirundo rustica*, 1 infected), chiffchaff (*Phylloscopus collybita*, 1), Eurasian blackcap (*Sylvia atricapilla*, 2), Eurasian reed warbler (*Acrocephalus scirpaceus*, 1), lesser whitethroat (*Sylvia curruca*, 1), sand martin (*Riparia riparia*, 1), spotted flycatcher (*Muscicapa striata*, 1) and willow warbler (*Phylloscopus trochilus*, 1).

If considering only these species, the prevalence was 1.3% (*n* = 686), and if considering all screened individuals belonging to these genera, the prevalence was 0.8% (*n* = 1037). The samples were either positive by PCR (two samples) or by cultivation (seven samples); in three cases, the alternative diagnostic method used in parallel revealed coinfection with another trypanosome lineage (lineages from the *T. everetti/bennetti* group twice and *T. culicavium* once). Only a single trypomastigote was found on all the inspected slides obtained from positive birds (Figure 3L).

#### Experimental infections of mosquitoes

**Figure 1.**
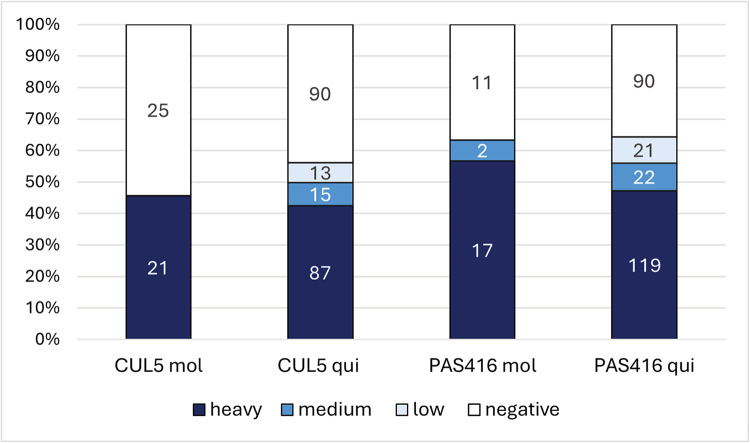
Infection rates and intensities in mosquitoes *Culex pipiens molestus* (mol) and *Culex pipiens quinquefasciatus* (qui) experimentally infected with *Trypanosoma tertium* n. sp. strains CUL5 and PAS416. Infection intensities: low 1–100 parasites; medium 100–1000 parasites; heavy >1000 parasites per gut. Numbers in columns represent the number of dissected individuals from relevant infection categories (heavy, medium, low, negative).

Two subspecies of *Culex pipiens – Cx. p. quinquefasciatus* and *Cx. p. molestus –* were fed on blood with two different strains of *T. tertium* n. sp. originating from two different hosts: CUL5 (mosquito) and PAS416 (bird). The CUL5 isolate was able to develop an infection in 45% of *Cx. p. molestus* and 55% of *Cx. p. quinquefasciatus,* with 100% prevalence of heavy infection among all infected *Cx. p. molestus* and 90% of heavy infection among all infected *Cx. p. quinquefasciatus.* Mosquitoes were also highly susceptible to the avian isolate, with approximately 65% prevalence in both *Cx. pipiens* subspecies and with 89% and 75% of heavy infections in *Cx. p. molestus* and *Cx. p. quinquefasciatus*, respectively (Figure 1).

To assess the vector specificity of *Culex* mosquitoes, 40 females of *Ae. aegypti* fed on blood with CUL5 were dissected. None of the females was found to be positive. Control mosquitoes, *Cx. p. quinquefasciatus* fed on the same inoculum, were infected in 60% of cases.

**Figure 2.**
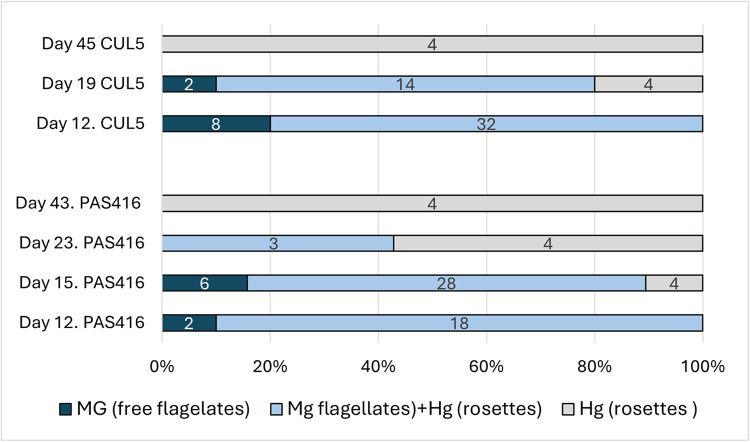
Temporal dynamics of trypanosomes localization in *Culex pipiens quinquefasciatus* guts experimentally infected by trypanosome isolates CUL5 and PAS416. Numbers of dissected females are shown in the columns. Mg, midgut; Hg, hindgut.

Trypanosomes in guts dissected 12 days post infection (dpi) were localized mainly in the midgut as unattached long epimastigotes along with rosettes in the hindgut, or exclusively in midgut as unattached epimastigotes (Figure 2). From 15 dpi onwards, the ratio of individuals with trypanosomes present only in the hindgut as rosettes increased slowly, while the proportion of infections in the hindgut and midgut or exclusively in the midgut decreased. At days 43–45, only rosettes were present, exclusively in the hindgut. Infections with rosettes in the midgut were not detected.

### Trypanosomes in diuretic liquid

**Figure 3.**
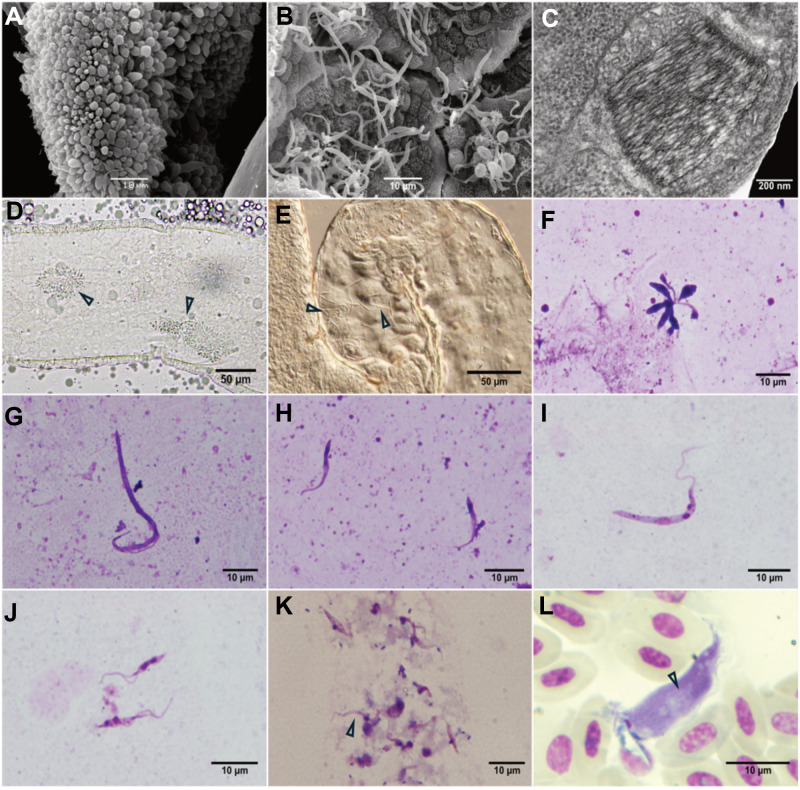
Scanning electron microscopy of *Trypanosoma tertium* n. sp. isolate CUL5 after experimental infection of *Culex pipiens quinquefasciatus*: rosette in hindgut (A), long epimastigotes in midgut (B). Transmission electron microscopy of the kinetoplast of *T. tertium* n. sp. from culture (C) (photo by L. Zídková). Dissected *Culex* mosquito gut infected by isolate CUL5: arrowheads pointing to the rosettes in hindgut (D) and long epimastigotes in midgut (E). Light microscopy of *T. tertium* n. sp. morphotypes in *C. quinquefasciatus* gut: rosette (F), long epimastigotes (G), short epimastigote (H); and morphotypes from culture: long epimastigote (I) and short epimastigotes (J). Short trypo/epimastigotes from diuretic liquid (K). Trypomastigote from barn swallow caught in Neuměřice, Czechia (L) with visible striation (see arrows); the white object partially covering the kinetoplast is an artefact.

Stages of *T. tertium* n. sp. were observed on 3 out of 7 Giemsa-stained droplets of diuretic liquid of examined mosquitoes. The expelled cell types were short trypo/epimastigotes (Figure 3K).

### Light microscopy

Three morphologically different types of trypanosomes were found in the guts of *Cx. p. quinquefasciatus* and *Cx. p. molestus* mosquitoes infected with *T. tertium* n. sp. isolates PAS416 and CUL5: long epimastigotes, short epimastigotes and short promastigotes forming rosettes (Figure 3A, B, D–H). In cultures, two different morphotypes were present: long and short epimastigotes (Figure 3I, J).

We used the analysis of variance to compare *T. tertium* n. sp. cell lengths based on the isolate (CUL5, PAS416), origin (gut, culture) and cell form (long or short epimastigote, rosette, diuresis form). The isolate as a factor had no significant impact (*F* = 3.86; *P* > 0.05), while both the origin and the cell form were significant (*F* = 101.19 and 315.40, respectively; *P* < 0.001). Therefore, the measurements of the two isolates were merged for the Tukey post hoc comparison test, which revealed that both gut and culture forms of short epimastigotes and rosettes are not significantly different from the diuresis form, while both long epimastigotes from the gut and culture differ from all remaining groups (see Table 2). Interestingly, cell lengths of long epimastigotes from cultures and guts are also significantly different.

**Table 2.** Morphometry of trypanosomes in mosquito gut and in culture

Cell type	*n*	Body length Mean ± S.D. (range)	Body width Mean ± S.D. (range)	Flagellum length Mean ± S.D. (range)	Tukey test for cell body length		
Long epimastigote gut	30	55.5 ± 5.5 (46.0–69.3)	2.1 ± 0.3 (1.2–2.5)	4.1 ± 1.5 (1.6–7.5)	*		
Long epimastigote culture	29	29.9 ± 3.1 (23.3–37.5)	1.8 ± 0.4 (1.6–3.2)	7.1 ± 2.5 (1.6–12.4)		*	
Short epimastigote gut	23	9.1 ± 0.9 (7.5–11.4)	1.5 ± 0.2 (1.2–1.9)	6.6 ± 0.8 (5.5–8.8)			*
Short epimastigote culture	22	10.7 ± 1.2 (8.3–14.1)	1.5 ± 0.2 (1.1–2.0)	7.5 ± 1.8 (1.6–9.8)			*
Rosette gut	44	9.0 ± 2.0 (5.2–13.9)	1.9 ± 0.5 (1.2–3.5)	5.4 ± 1.9 (1.3–8.2)			*
Diuresis	11	9.5 ± 0.5 (9.0–10.4)	1.3 ± 0.1 (1.1–1.6)	6.6 ± 0.6 (5.8–7.9)			*
Trypomastigote	1	32, 4	6	No free flagellum			

Values in micrometres. Asterisks in the same column indicate no significant differences in the cell length.

### Inoculation of experimental birds

Laboratory birds (canaries and zebra finches) were inoculated with mosquito guts infected with CUL5 or PAS416, respectively. Birds were inoculated perorally, subcutaneously (CUL5, PAS416) or transconjunctivally (PAS416) with heavily infected guts.

The isolate CUL5 was used for peroral inoculations of 1 zebra finch and 5 canaries. The isolate PAS416 was used for 8 peroral and 8 subcutaneous inoculation of canaries, 2 subcutaneous inoculations of zebra finches and 2 transconjunctival inoculations of canaries. All birds remained negative till the end of the experiment (6 months).

## Discussion

Despite advances in the last decade, our knowledge of vectors associated with avian trypanosomes and modes of their transmission still has gaps. The spectrum of avian trypanosome vectors includes members of multiple dipteran families with mosquitoes as vectors of three species: *T. culicavium* (Votýpka et al., 2012; Zídková et al., 2012), *T. thomasbancrofti* (Zídková et al., 2012; Šlapeta et al., 2016; Fialová et al., 2021) and *T. tertium* n. sp., described in this study.

Based on phylogenetic studies (SSU rRNA), it is evident that *T. tertium* n. sp. is a unique and well-supported monophyletic lineage of avian trypanosomes. It is closely related to *T. thomasbancrofti*, and both fall into the *T. avium* s. l. group C of avian trypanosomes. Moreover, the existence of these lineages was supported also by Random Amplified Polymorphic DNA (RAPD) analysis (Zídková et al., 2012). These findings reinforce their distinct phylogenetic position and underscore its evolutionary divergence from related taxa. The SSU rRNA sequence comparison of *T. tertium* n. sp. (CUL5) with its closest relative, *T. thomasbancrofti*, using the BLAST algorithm revealed 99.4% identity with the first hit of *T. thomasbancrofti* (CUL15), while there were 13 differences in a 2163 bases-long segment.

Trypanosoma thomasbancrofti and *T. tertium* n. sp. even share some characteristics, e.g., mature infections are localized in the vector’s hindgut, similarly to other members of the group whose proven vectors are blackflies (group C lineages X and XI). On the contrary, the *T. culicavium* lineage belongs to group B of avian trypanosomes and is localized on the stomodeal valve of the vector (Votýpka et al., 2012; Zídková et al., 2012). However, *T. corvi*, a species transmitted by hippoboscid flies and belonging to the same group B as *T. culicavium*, has infectious stages localized in the hindgut. That points out the fact that although the localization is important for the ways of transmission, it is not related to the phylogenetic position of the parasite.

Apart from *T. culicavium* and *T. thomasbancrofti*, two recently described avian trypanosomes infecting *Culex* mosquitoes (Votýpka et al., 2012; Zídková et al., 2012; Fialová et al., 2021), other kinetoplastids in *Culex* mosquitoes have been reported as early as the beginning of the 20th century, and it is crucial to distinguish *T. tertium* n. sp. from these previously described species to confirm that it represents a truly new species. *Trypanosoma noctua*, described by Schaudinn (1904), was reported to infect *Culex* mosquitoes after feeding on an infected little owl (*Athene noctua*). However, it is likely that Schaudinn worked with mosquitoes naturally infected with monoxenous kinetoplastids from the genus *Crithidia* (see Novy et al., 1907) rather than with any avian trypanosome, including *T. tertium* n. sp. A second species reported from *Cx. pipiens* mosquitoes is *T.* (*Herpetomonas*) *culicis* (Novy et al., 1907). These authors described this trypanosome from the intestines of naturally infected mosquitoes and reported two morphological forms. ‘Very long forms’ measured 30–35 µm, while ‘short forms’ ranged between 12 and 20 µm. The long epimastigotes of *T. tertium* n. sp. documented in this study measured 55 µm on average, almost twice as much; such a difference is substantial. (Novy et al., 1907) also described fast movement of long forms from the gut, which made them difficult to follow. We did not observe such a fast movement of long epimastigotes of *T. tertium* n. sp. The size of short epimastigotes (average 9–11, maximum 14 µm) overlaps with that of *T. culicis* short epimastigotes; however, short forms in the intestine overlapping in length can be found in many other trypanosome species belonging to all three groups (Zídková et al., 2012). In addition, Novy et al. (1907) do not mention any stages similar to the rosette forms found in the intestines of mosquitoes infected by *T. tertium* n. sp. For these reasons, we consider *T. culicis* as a separate species from *T. tertium* n. sp. In 1961, *T. culicis* was transferred to the genus *Blastocrithidia* by Wallace and Johnson (1961). However, we consider this inappropriate since the authors did not work with the original isolate; on the contrary, they used their own isolate originating from a different mosquito species and genus, *Aedes vexans*. During our studies, we failed to find any *Aedes* mosquitoes infected by *T. tertium* n. sp. despite a substantial number of examined specimens (959 individuals in this study, 1398 individuals in Brotánková et al., 2022). Furthermore, there is a considerable degree of host specificity of mosquito trypanosomes, since neither *T. culicavium* nor *T. thomasbancrofti* were ever detected in *Aedes* mosquitoes (Fialová et al., 2021; Brotánková et al., 2022). Moreover, *Aedes* mosquitoes were not susceptible to the infection by *T. tertium* n. sp. in our experiments, nor to *T. culicavium* (Votýpka et al., 2012). It is therefore unlikely that different mosquito genera would share trypanosomes including *T. tertium* n. sp. This applies not only to avian trypanosomes transmitted by mosquitoes but also to hippoboscid/avian lineages as well as to mammalian *T. theileri* trypanosomes (Brotánková et al., 2022; Santolíková et al., 2022).

Mosquitoes naturally infected with trypanosomes that molecularly match *T. tertium* n. sp. have been found in Czechia for the first time in 1999/2000 (Zídková et al., 2012); from 28 strains established from 898 examined *Cx. pipiens* guts, 2 belonged to *T. tertium* n. sp. (prevalence 0.22%, Svobodová et al., 2015). In our study, only 1 individual out of 1128 dissected *Cx. pipiens* mosquitoes harboured *T. tertium* n. sp. (prevalence 0.08%). The prevalence of *T. tertium* n. sp. was comparable to that of *T. thomasbancrofti* but notably lower than the prevalence of *T. culicavium* (3.1 %), which was similar to previous findings (Svobodová et al., 2015). Given its low prevalence and the historical under-representation of avian trypanosomes in research, it is more likely that the absence of earlier records reflects a lack of detection rather than a recent introduction. Moreover, the prevalence of *T. tertium* n. sp. among wild mosquitoes is so low that studies with less investigated mosquitoes can fail to detect it at all, like the study from Lithuania with 561 dissected *Cx. pipiens* females (Valavičiūtė-Pocienė et al., 2024).

The prevalence among avian hosts was likewise low, with only 9 (0.3%) out of the 3084 screened passerines found to be infected by *T. tertium* n. sp. Since the mode of transmission of *T. tertium* n. sp. is probably similar to *T. culicavium* and *T. thomasbancrofti*, i.e., after ingestion of the infectious vector, it is appropriate to focus on insectivorous avian species when assessing prevalence, in which the prevalence of *T. tertium* n. sp. rises to 1.3%. In our previous studies in the years 2002–2007, using cultivation as a diagnostic method, 207 out of 722 passerines were positive for trypanosomes; 105 isolated were established in culture, and just 4 belonged to *T. tertium* n. sp. Besides the predominantly insectivorous species, the collared flycatcher (*Ficedula albicollis*, 1 infected), the chiffchaff (*Phylloscopus collybita*, 2 infected) and 1 reed warbler (*Acrocephalus scirpaceus*; Musa et al., 2024) *T. tertium* n. sp. was found in one nuthatch (*Sitta europaea*; Černý, 2006; Szabová, 2008; Zídková et al., 2012).

On all the inspected slides originating from positive birds, only a single trypomastigote of *T. tertium* n. sp. was found. The morphological differentiation of avian trypomastigotes is challenging and requires an experienced observer. However, certain morphological traits of trypomastigotes can provide clues for classification – usually not at the species level, but rather at the group level – such as the presence of longitudinal striations (Kostygov et al., 2021). Moreover, avian trypanosomes occur in the peripheral blood of birds at low prevalence, making it difficult to detect trypomastigotes microscopically as was the case for *T. tertium* n. sp. in our study (one trypomastigote found). The *T. tertium* n. sp. trypomastigote from the naturally infected barn swallow had evident longitudinal striations, comparable to *T. thomasbancrofti* and characteristic for trypanosomes belonging to the group C (Šlapeta et al., 2016; Fialová et al., 2021; Kostygov et al., 2021).

Despite the low prevalence of avian trypanosomes transmitted by mosquitoes, their occurrence is considered transcontinental since *T. thomasbancrofti* was found in mosquitoes and birds in Europe as well as in regent honeyeaters (*Anthochaera phrygia*) in Australia (Zídková et al., 2012; Šlapeta et al., 2016). The same applies to *T. tertium* n. sp.; sequences from this parasite were detected in Europe as well as in the Australian mudlark (*Grallina cyanoleuca*) in Australia (Zídková et al., 2012; Cooper et al., 2017; Musa et al., 2024). It is possible that the distribution of all avian trypanosome species is worldwide, and the lack of reports of the mosquito-transmitted species from other continents is caused by insufficient sampling. Although *T. avium* s. s. and *T. bennetti/T. everetti* were found in Africa and America (Valkiūnas et al., 2016; Galen et al., 2020; Magaña Vázquez et al., 2022), the mosquito species remained undetected.

For experimental confirmation of the vectorial role of mosquitoes in the *T. tertium* n. sp. life cycle, laboratory mosquitoes were infected with two isolates originating from both host types (mosquito, bird). The mosquito as well as the avian isolates were able not only to infect both *Cx. p. pipiens* subspecies but also developed high prevalences and infection intensities similar to experimental infections with the other 2 mosquito species (Votýpka et al., 2012; Fialová et al., 2021). Experimental mosquitoes were dissected after defaecation, confirming the specificity of the infection. *Culex* mosquitoes thus have a high capacity to sustain the development of *T. tertium* n. sp.

The localization of *T. tertium* n. sp. in the gut after blood digestion changes with time. Initially, the majority of mosquitoes harboured long epimastigotes in the midgut and rosettes in the hindgut. In the course of infection, midgut stages disappeared, resulting in rosettes localized exclusively in the hindgut, which is typical for group C of avian trypanosomes and is consistent across multiple vectors (Votýpka and Svobodová, 2004; Svobodová and Rádrová, 2018; Fialová et al., 2021). The localization of trypanosomes within the gut of infected mosquitoes exhibits notable differences among species transmitted by mosquitoes. Mature *T. culicavium* infections are localized exclusively on the stomodeal valve (Votýpka et al., 2012). In contrast, *T. thomasbancrofti* typically forms rosettes in the hindgut but can be observed in the midgut at later stages of the infection as short epimastigotes (Fialová et al., 2021). However, *T. thomasbancrofti* epimastigote bodies measure only 8.7 µm on average and are therefore many times shorter than midgut epimastigotes of *T. tertium* n. sp. (55.5 µm), which are moreover present in the midgut from the very beginning of infection. Some studies also described midgut localization of mosquito trypanosomatids, but these observations were from initial phases of infections, when the peritrophic matrix was still present (Bennett, 1961, 1970; Chatterjee, 1977). Moreover, trypanosomes transmitted by mosquitoes can be distinguished not only by their localization in the gut but also by their dimensions and the thickness of the kinetoplast (Zídková et al., 2012). For a comparison of dimensions of *T. tertium* n. sp. with other trypanosome species transmitted by mosquitoes, see Table 3. However, identification of avian trypanosomes from vectors based solely on morphology – without knowledge of the vector and without information on localization within the gut – is challenging. The morphological features, as cell size ranges can overlap (Zídková et al., 2012), and multiple developmental forms can be found in the gut of vectors. Molecular techniques are key to accurately identifying the species, as they provide the necessary resolution that morphology alone cannot achieve.

**Table 3.** Dimensions of whole cells of trypanosomes known to infect *Culex* mosquitoes (average, (range))

Species	Long epimastigote gut (µm)	Long epimastigote culture (µm)	Short epimastigote gut (µm)	Short epimastigote culture (µm)	Kinetoplast height (nm)	Blood trypomastigote (µm)	References
*T. tertium* n. sp.	59.6 (47.6–76.8)	37 (24.9–40.7)	15.7 (14–20.2)	17.6 (9.9–23.9)	749–859* (579–966)	32.4	This study *Zídková et al., 2012
*T. thomasbancrofti*	n. p.	17.8–20.4 (13.0–29.9)	16.5 (7.5–27.8)	10.1–10.5 (7.3–13.9)	527–546 (446–668)	38.3	Zídková et al., 2012 Šlapeta et al., 2016 Fialová et al., 2021
*T. culicavium*	24.3	27.8–30.8 (18.5–50.6)	12.0	13.1–13.7 (9.8–16.7)	295–341 (219–423)	40.7, 40.9	Zídková et al., 2012 Votýpka et al., 2012
*T. culicis*	30–45	35–40	10–23	12–15	n. a.	n. a.	Novy et al., 1907

n. p., not present; n. a., not analysed.

To achieve transmission, several birds were inoculated. Based on the localization of *T. tertium* n. sp. in the midgut and hindgut of mosquitoes along with its presence in the diuretic liquid, three distinct inoculation methods were tested based on previous studies: peroral, transconjunctival and subcutaneous (Votýpka and Svobodová, 2004; Svobodová et al., 2017; Svobodová and Rádrová, 2018; Fialová et al., 2021). Transmission by mosquito bite is unlikely, as even *T. culicavium*, which is localized on the stomodeal valve, is not transmitted this way (Votýpka et al., 2012). Despite substantial effort, transmission to experimental birds was not successful. However, *T. tertium* n. sp. has not been identified in bloodsucking Diptera other than *Culex* mosquitoes (Zídková et al., 2012; Svobodová et al., 2015; Brotánková et al., 2022, and unpublished data) and is highly infective to laboratory mosquitoes, which strongly suggests mosquitoes as natural vectors of *T. tertium* n. sp. Canaries and zebra finches used in this study might not represent the best model hosts; indeed, previous transmission experiments also yielded somewhat different outcomes based on the parasite species used. In most cases, transmission was achieved through oral or conjunctival inoculation (Votýpka and Svobodová, 2004; Votýpka et al., 2012; Svobodová and Rádrová, 2018; Fialová et al., 2021). However, in the case of *T. everetti/bennetti*, transmission only succeeded after subcutaneous inoculation. It seems thus probable that trypanosomes differ in their avian host specificity, resulting in different prevalences in wildlife.

In conclusion, we describe a new species of avian trypanosome, *T. tertium* n. sp., whose probable vector is *Cx. pipiens*. Trypanosomes isolated from both the vector and the avian host developed high prevalences and parasitaemias in experimentally infected mosquitoes, and putative infectious stages were observed in the hindgut as well as in the diuretic liquid, suggesting transconjunctival transmission in addition to vector ingestion. Prevalences of *T. tertium* n. sp. in nature are low both in vectors and birds when compared to other avian trypanosomes including those with mosquito vectors. This might be related to a narrower range of hosts since only insectivorous birds are potentially exposed to the mosquito-dwelling parasite.

## Taxonomic summary

**ZooBank registration of this work**: urn: lsid:zoobank.org: pub:995C568B-8479-44CE-978D-11B21CE148C1.

**Taxonomic assignment**: Discoba: Discicristata: Euglenozoa: Kine- toplastea: Trypanosomatida: Trypanosomatidae: *Trypanosoma*

*Trypanosoma tertium* n. sp.

**Diagnosis:** *Trypanosoma* with three different morphotypes in mosquito host: long epimastigotes, 55.5 ± 5.5 µm long and 2.1 ± 0.3 µm wide with a free flagellum 4.1 ± 1.5 µm long; short epimastigotes, 9.1 ± 0.9 long, 1.5 ± 0.2 wide and a free flagellum 6.6 ± 0.8 µm long, rosette forms 9.0 ± 2.0 µm, long 1.9 ± 0.5 µm wide with a flagellum 5.4 ± 1.9 µm long.

The kinetoplast is 0.310 ± 0.031 µm thick (Figure 3C) (Zídková et al., 2012, Table 2).

Two different morphotypes are present in culture: long epimastigotes, 29.9 ± 3.1 long, 1.8 ± 0.4 µm wide with 7.1 ± 2.5 µm long flagellum; short epimastigotes, 10.7 ± 1.2 µm long, 1.5 ± 0.2 µm wide with flagellum 7.5 ± 1.8 µm long.

Trypanosomes expelled during diuresis are in the form of trypo-/epimastigotes, 9.5 ± 0.5 µm long, 1.3 ± 0.1 µm wide and with a flagellum 6.6 ± 0.6 µm long.

Trypomastigote narrow, with a tapering posterior end and visible longitudinal striation. An undulating membrane is located on the external side of the curvature. The cell body is approximately 32 µm long and 6 µm wide.

**Type locality**: Czechia, South Moravia, Milovice Forest (48.8213 N, 16.6932 E).

**Type isolate**: CUL5 was isolated by Milena Svobodová and is deposited in the collection of the Department of Parasitology, Faculty of Science, Charles University, Prague, Czechia.

**Type material**: 11 permanent slides with Giemsa-stained syntypes of *T. tertium* n. sp. deposited in the collection of the National Museum of the Czech Republic, inventory numbers P6E 5584-P6E 5595 (Table 4). All cells on the slides are syntypes. Some syntypes are shown in Figure 3F–J.

**Table 4.** Overview of deposited samples containing specimens of *T. tertium* n. sp

Sample	Inventory no.	Strain	Origin of the sample
1	P6E5584	Pas416	Exp. infection – *Cx. p. quinquefasciatus* gut
2	P6E5585	Pas416	Exp. infection – *Cx. p. quinquefasciatus* gut
3	P6E5586	Pas416	Exp. infection – *Cx. p. quinquefasciatus* gut
4	P6E5587	Pas416	Culture
5	P6E5588	Pas416	Culture
6	P6E5589	Pas416	Culture
7	P6E5590	Cul5	Exp. infection – *Cx. p. quinquefasciatus* gut
8	P6E5591	Cul5	Exp. infection – *Cx. p. quinquefasciatus* gut
9	P6E5592	Cul5	Exp. infection – *Cx. p. quinquefasciatus* gut
10	P6E5593	Cul5	Culture
11	P6E5594	Cul5	Culture
12	P6E5595	Cul5	Culture

**Type host (vector)**: Common house mosquito *Culex pipiens* (Insecta: Diptera, Culicidae).

**Additional hosts (avian)**: Insectivorous songbirds (Passer- iformes): barn swallow *Hirundo rustica* (Linnaeus, 1758), collared flycatcher *Ficedula albicollis* (Temminck, 1815), common chiffchaff *P. collybita* (Vieillot, 1817), Eurasian blackcap *Sylvia atricapilla* (Linnaeus, 1758), Eurasian nuthatch *Sitta europaea* (Linnaeus, 1758), Eurasian reed warbler *Acrocephalus scirpaceus* (Hermann, 1804), lesser whitethroat *Sylvia curruca* (Linnaeus, 1758), sand martin *Riparia riparia* (Linnaeus, 1758), spotted flycatcher *Muscicapa striata* (Pallas, 1764) and willow warbler *Phylloscopus trochilus* (Linnaeus, 1758).

**Gene sequence**: An SSU rRNA gene sequence is deposited in GenBank with accession number JN006838.

**Etymology**: Epithet. L. n. adj. *tertium* (third). The name relates to the fact that this is the third *Trypanosoma* species, which is described from mosquitoes and characterized molecularly. Besides, the organism was originally designated as lineage III in group C of avian trypanosomes (Zídková et al., 2012).
